# Inferring functional connectivity through graphical directed information

**DOI:** 10.1088/1741-2552/abecc6

**Published:** 2021-03-30

**Authors:** Joseph Young, Curtis L Neveu, John H Byrne, Behnaam Aazhang

**Affiliations:** 1 Department of Electrical & Computer Engineering, Rice University, Houston, TX 77005, United States of America; 2 Department of Neurobiology & Anatomy, McGovern Medical School of The University of Texas Health Science at Houston, Houston, TX 77030, United States of America

**Keywords:** information theory, causality, functional connectivity, aplysia, directed information, mutual information, indirect connectivity

## Abstract

**Objective.:**

Accurate inference of functional connectivity is critical for understanding brain function. Previous methods have limited ability distinguishing between direct and indirect connections because of inadequate scaling with dimensionality. This poor scaling performance reduces the number of nodes that can be included in conditioning. Our goal was to provide a technique that scales better and thereby enables minimization of indirect connections.

**Approach.:**

Our major contribution is a powerful model-free framework, graphical directed information (GDI), that enables pairwise directed functional connections to be conditioned on the activity of substantially more nodes in a network, producing a more accurate graph of functional connectivity that reduces indirect connections. The key technology enabling this advancement is a recent advance in the estimation of mutual information (MI), which relies on multilayer perceptrons and exploiting an alternative representation of the Kullback–Leibler divergence definition of MI. Our second major contribution is the application of this technique to both discretely valued and continuously valued time series.

**Main results.:**

GDI correctly inferred the circuitry of arbitrary Gaussian, nonlinear, and conductance-based networks. Furthermore, GDI inferred many of the connections of a model of a central pattern generator circuit in *Aplysia*, while also reducing many indirect connections.

**Significance.:**

GDI is a general and model-free technique that can be used on a variety of scales and data types to provide accurate direct connectivity graphs and addresses the critical issue of indirect connections in neural data analysis.

## 1. Introduction

Extracting the functional connectivity among a network of neurons has remained a daunting challenge in systems neuroscience. A variety of techniques [1–6] have addressed the *causality* present within connectivity and accordingly assign directionality to connections. For example, some research [1, 5] has focused on causality among fine-scale neuronal networks where binned spike times are used to determine directed functional connectivity between individual neurons. In contrast, other research has taken a large-scale approach by inferring directed functional connectivity between brain regions via electrocorticography (ECoG) [2–4] and electroencephalography (EEG) [6] recordings. In either case, progress has been hindered significantly by the inability to fully condition model-free connectivity inferences on the activity of the other nodes within the network being analyzed, the consequence of which is analyses that may include indirect connections. Prior work has included applying model assumptions such as Gaussianity [6] or conditioning on only one other node [1]. A better way to realize directed information (DI) graphs [7] is to develop an estimator that is model-free and that scales well with dimensionality so that conditioning can be applied to many nodes in a graph. Therefore, our first major goal was to develop a framework that produces directed functional connectivity inferences that are conditioned on many of the nodes in the network and are completely free of any model assumptions. Importantly, conditioning means that our connectivity analyses result in a graph [7] where connectivity more accurately expresses conditional dependence rather than dependence contaminated by other nodes’ activity, meaning that these graphs preserve direct connections and minimize indirect connections. Our second goal was to develop a framework which can easily be applied at any scale of recordings ranging from spike times to voltage recordings.

DI [3, 5, 8, 9] is a useful tool in quantifying directed functional connectivity, primarily because it has no inherent model assumptions. This feature is critical in the case of neural data, which is unlikely to be non-Gaussian. Accordingly, DI and transfer entropy, which can be parameterized to be equivalent to DI, have gained traction in the neuroscience community [10–12]. However, estimation of DI and transfer entropy has been plagued by the dimensionality issues, which have been a key limiting factor preventing the conditioning of directed functional connectivity estimates on the entire graph. For example, one study on ECoG only considered pairwise DI [2], whereas others on ECoG [3] and spike times [1] at best considered conditioning on one single other node. Recently, advances [13, 14] in the estimation of mutual information (MI) between high dimensional variables have been achieved and these approaches have already led to the estimation of pairwise directed functional connectivity in DI [15] and transfer entropy [16]. However, no work to our knowledge has been done on using this estimator to condition directed functional connectivity estimates on other nodes in a network to produce a graph [7]. We leverage this approach by introducing graphical DI (GDI), which builds on prior DI graph work [7] by incorporating this estimation technique to overcome previous limitations preventing model-free conditioning of DI estimates on many other nodes.

Although previous DI tools have generally focused on either discretely valued time series [1, 5, 17] such as binned spike times (taking on the values 0 or 1) or continuously valued time series such as voltages obtained from ECoG [2–4] or EEG [6], our framework that is based on a prior conditional MI estimator [14] can be universally applied to either type of data. This universality is because likelihood ratio estimation is at the heart of our estimation approach, which means it is independent of the nature of the likelihoods. In particular, using this ratio provides abstraction from whether likelihoods are based on probability density functions for continuously valued data or based on probability mass functions for discretely valued data. Therefore, whether data consists of spikes, ECoG recordings, or another modality, it can be easily inserted into our estimator to produce a DI graph.

To begin, we present background on DI, introduce GDI, and describe how to estimate such quantities as well as how to perform sign inference in regards to whether a connection is excitatory or inhibitory. Then, we present a series of results demonstrating the necessity of a graphical method such as GDI in assessing connectivity. We begin with two continuously-valued simulations consisting of 11 nodes as an analog to ECoG or EEG voltage time series data. The first simulation is a Gaussian case where the analytic solution is known and can be compared with our GDI estimates. Because neural data is unlikely to be Gaussian or have linear relationships, we follow our Gaussian analysis with a nonlinear network analysis that shows GDI’s generality. We continue with results on discretely-valued data in the form of simulated binned spike times from 11 and 13 node networks of conductance-based neurons. Finally, we discuss how our findings demonstrate the power and flexibility of GDI.

## 2. Methods

### 2.1. Directed information

DI [3, 5, 8, 9] is an information theoretic tool that measures the causal influence of one entity upon another without any model constraints. In order to define DI, we begin by defining the MI between two continuous random variables *X* and *Y* [18]:

(1) $$I(X;Y)=D_{KL}\left(p_{X,Y}∥p_{X}p_{Y}\right)$$

(2) $$=\int_{S_{Y}}\int_{S_{X}}p_{X,Y}(x,y)\text{ log }\left(\frac{p_{X,Y}(x,y)}{p_{X}(x)p_{Y}(y)}\right)\text{dxdy},$$

where *D*_*KL*_ is the Kullback–Leibler (KL) divergence, *p*_*X*,*Y*_ is the joint probability density associated with *X* and *Y*, *p*_*X*_ and *p*_*Y*_ are the marginal probability densities for *X* and *Y*, and finally *S*_*X*_ and *S*_*Y*_ are the support of *X* and *Y* where *p*_*X*_(*x*) > 0 and *p*_*Y*_(*y*) > 0. Importantly, MI quantifies the statistical dependence between *X* and *Y*, including when they are non-Gaussian or discrete. We also note that MI can be defined in terms of differential entropies *h* [18]:

(3) I(X;Y)=h(X)+h(Y)−h(X,Y),

where $h(X)=-\int_{S_{X}}p_{X}(x)\text{ log }p_{X}(x)dx$, $h(Y)=-\int_{S_{Y}}p_{Y}(y)\text{ log }p_{Y}(y)dy$, and $h(X,Y)=-\int_{S_{X},S_{Y}}p_{X,Y}(x,y)\text{ log }p_{X,Y}(x,y)\text{dxdy}$ [18].

Importantly, MI can be conditioned on other random variables, such as *W*, to produce Conditional MI (CMI) [18]:

(4) I(X;Y∣W)=I(X;Y,W)−I(X;W).

The DI from the random process **X** to the random process **Y** can then be defined as a particular formulation of conditional MI [3, 8, 19–21]:

(5) $$I(X\to Y)=I\left(X_{i-M},\ldots ,X_{i-1};Y_{i}|Y_{i-M},\ldots ,Y_{i-1}\right),$$

where *X*_*i*_ and *Y*_*i*_ refer to the *i*th samples of **X** and **Y**, respectively, and *M* is the memory parameter that refers to how many past samples in both processes affect a given current value of **Y**. In essence, DI is the MI between past values of **X** and the current value of **Y** at any given time *i* conditioned on **Y**’s own past to just capture causal influence from **X** to **Y**.

### 2.2. Graphical directed information (GDI)

**Figure 1.**
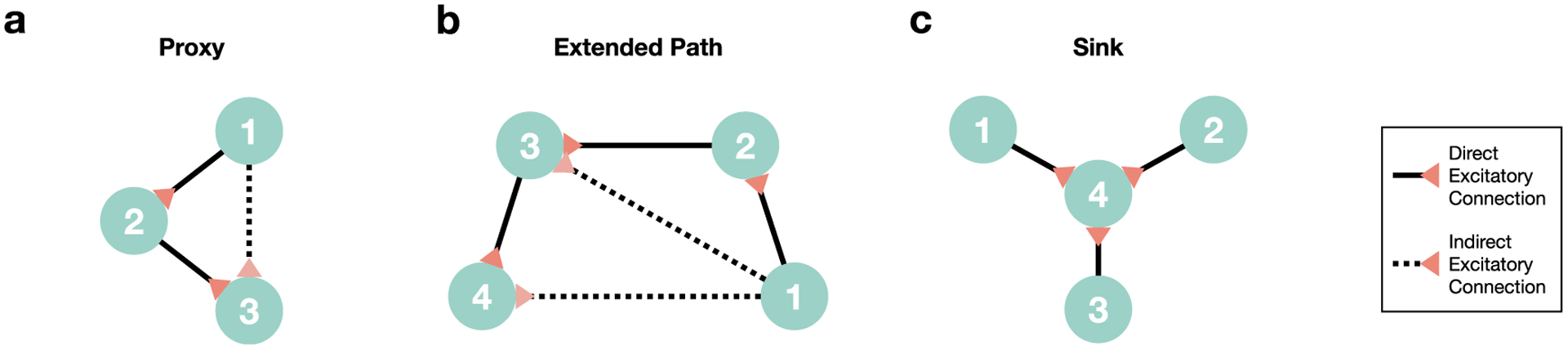
Examples of motifs addressed by graphical methods such as GDI. (a) In the case of node 1 directly influencing node 2 and node 2 directly influencing node 3, an indirect proxy connection from node 1 to node 3 will be detected by GDI and eliminated by conditioning on the activity of node 2. (b) In the case of an extended path from node 1 to node 4, simple pairwise analyses will catch two indirect connections. In contrast, GDI conditions analyses on all other nodes’ activity and therefore the indirect connections will be eliminated. (c) In the case of a sink, i.e. where multiple nodes influence one node, simple pairwise analyses may not detect direct connections because of thresholding methods that are often used in analyzing graphs. Because three connections to node 4 exist, each connection will appear to have a small influence on node 4 that may be sub-threshold. GDI’s conditioning on other edges will increase the relative contribution of a given direct edge, pushing it above threshold and allowing it to be detected.

Equation (5) can be extended to condition not only on the past of **Y** but on the past of other random processes as well. The motivation for performing such conditioning is to be able to identify and eliminate indirect relationships which can occur as a result of the network motifs displayed in figure 1. For example, a *proxy* connection [5] (figure 1(a)) occurs when two nodes appear to be connected because of an intermediate third node. Additional intermediate nodes can create an *extended path* (figure 1(b)) where multiple indirect connections appear along the path. However, conditioning can do more than eliminate the indirect connections present in these motifs. For the case of a *sink* (figure 1(c)), which is a node influenced by multiple other nodes, conditioning increases the strength of connections to the sink which has the advantage of making such direct connections more likely to be detected.

In order to define our graphical methodology, which addresses such network motifs, we first consider the case where **X** and an additional random process **W** both influence **Y**. We can capture the effect that **X** has on **Y** by accounting for the influence of **W** on **Y** in the form of conditional DI (CDI) [3]:

(6) $$I(X\to Y|W)=I(X_{i-M},\ldots ,X_{i-1};Y_{i}|Y_{i-M},\ldots ,Y_{i-1},W_{i-M},\ldots ,W_{i-1}).$$

To take this concept to the limit and consider an entire graph, we now change our notation by replacing the three lettered random processes **X**, **Y**, and **W** with *R* indexed random processes **X**^(1)^, **X**^(2)^, …, **X**^(**R**)^, which can each be considered nodes in the graph. As in prior work [7], considering DI for this network results in what we refer to as GDI:

(7) $$I_{G}\left(X^{\left(j\right)}\to X^{\left(k\right)}\right)=I\left(X^{\left(j\right)}\to X^{\left(k\right)}|X^{\left(*\right)}\right),$$

where **X**^(^*^)^ = {**X**^(1)^, **X**^(2)^, …, **X**^(**R**)^} \ {**X**^(**j**)^, **X**^(**k**)^}.This formulation allows DI to be implemented in a fashion true to graph theory, where an edge from node *j* to node *k* represents conditional dependence quantified by *I*_*G*_(**X**^(**j**)^ → **X**^(**k**)^). Finally and formally, similar to [7], we define a DI graph as a directed weighted graph *G*:

(8) G=(V,E,w)

(9) $$V=\left\{v^{\left(1\right)},v^{\left(2\right)},\ldots ,v^{\left(R\right)}\right\}$$

(10) $$E=\left\{(j,k):j,k\in V\text{ and }w^{\left(j,k\right)}>0\right\}$$

(11) $$w^{\left(j,k\right)}=S^{\left(j,k\right)}\times I_{G}\left(X^{\left(j\right)}\to X^{\left(k\right)}\right),$$

where *V* denotes the set of nodes, *E* denotes the set of edges, *w*^(*j*,*k*)^ defines the edge weights as signed GDI, and *S*^(*j*,*k*)^ is formally defined later in (19) as the sign for GDI and is either −1 to indicate inhibition or +1 to indicate excitation. In the next subsection, we consider a new GDI estimation approach.

### 2.3. GDI estimation

DI has been estimated via a variety of data-driven methods in the past such as an empirical plug-in method [7], context-tree weighting (CTW) [17, 22], context-tree maximizing (CTM) [1, 23, 24], kernel density estimation (KDE) [3, 25], and *k*-nearest neighbors (*k*-NN) [2, 26]. Part of the reason for this variety is due to the different ways that DI can be defined: CTW, CTM, and KDE, as well as the plug-in method, use probability distributions, *k*-nn uses entropies, and for our work we use an alternative representation of the KL-divergence [14, 27]. Critically, the CTW, CTM, KDE, and *k*-nn methods of DI estimation are known to scale poorly with dimensionality [1, 14]. Prior studies using CTM [1], KDE [3], and *k*-nn [2] commented on this scaling difficulty, and in practice only conditioned on at best one other node. Importantly, implementation of DI estimation via the KL-divergence has been shown to scale very well with dimensionality in the continuously valued case [14] even compared to *k*-nn, and our scaling results presented later indicate strong scaling performance for the discretely valued case as well.

The two key features that allow for this leap in DI estimation are the exploitation of an alternative representation of the KL-divergence [13, 27] combined with multi-layer perceptrons’ strong classification performance [14]. Notably, DI can be represented as the difference of two MI terms (4) which can each be represented in terms of KL-divergences (1). Therefore, we begin by showing the Donsker-Varadhan representation of the KL-divergence which enables the use of a classifier for its estimation [13, 14, 27]:

(12) $$D_{KL}\left(p_{X}∥q_{X}\right)=\underset{f\in F}{\text{sup }}[\underset{x\sim p_{X}(x)}{E}[f(x)]-\text{log }\left(\underset{x\sim q_{X}(x)}{E}[\text{exp}(f(x))]\right)],$$

where F is a function class defined to consist of the functions that produce finite expectations. It turns out that the optimal function is known [13, 14] to be the point-wise log-likelihood ratio $f^{*}(x)=\text{log}\frac{p_{X}(x)}{q_{X}(x)}$, and therefore this ratio is estimated from data. Prior work [14] found estimation of this ratio via a multi-layer perceptron trained with a binary cross entropy loss function to outperform a gradient boosted decision tree approach in estimating the KL-divergence when ultimately estimating CMI. Therefore, we use this approach to ultimately estimate DI because it is a particular formulation of CMI. Considering that one has *N*_*p*_ samples from *p*_*X*_(*x*) and *N*_*q*_ from *q*_*X*_(*x*) with individual samples referred to respectively as *x*_*n*,*p*_ and *x*_*m*,*q*_, a binary classifier implemented via a neural network can be trained to identify data from *p*_*X*_(*x*) as label *ℓ* = 1 and data from *q*_*X*_(*x*) as label *ℓ* = 0 [14, 28, 29]. Intuitively, if *p*_*X*_(*x*) and *q*_*X*_(*x*) are different, then classification accuracy should deviate from the random guess accuracy of 0.5. For a given point *k*, the point-wise likelihood ratio can then be computed as $L\left(x_{k}\right)=\frac{P\left(\ell =1|x_{k}\right)}{1-P\left(\ell =1|x_{k}\right)}$ and this can substituted into (12) to produce an estimate of the KL-divergence [14]:

(13) $${\hat{D}}_{KL}\left(p_{X}∥q_{X}\right)=\frac{1}{N_{p}}\overset{N_{p}}{\underset{n=1}{\sum }}\text{log}L\left(x_{n,p}\right)-\text{log }\left(\frac{1}{N_{q}}\overset{N_{q}}{\underset{m=1}{\sum }}L\left(x_{m,q}\right)\right).$$

In essence, the estimates of the point-wise likelihood ratio coming from a neural network applied to the data are plugged into a series of equations ((13), (1), and (4)) to estimate DI and similarly GDI, which is elaborated in the final paragraph of this subsection.

We use the implementation provided by prior work (https://github.com/sudiptodip15/CCMI) [14] which bootstraps classifiers. One bootstrap iteration consists of randomly selecting two-thirds of the data to be used for training the classifier, while the remaining one-third of data is used for testing the classifier. The test data corresponds to the *N*_*p*_ samples from *p*_*X*_(*x*) and *N*_*q*_ from *q*_*X*_(*x*) referred to previously. Additional bootstrap iterations each randomly split the data between training and testing sets, and a final ${\hat{D}}_{KL}\left(p_{X}∥q_{X}\right)$ estimate is obtained by averaging across the individual ${\hat{D}}_{KL}\left(p_{X}∥q_{X}\right)$ estimates obtained via each bootstrap iteration. Increasing the number of bootstrap iterations leads to a more reliable estimate.

**Figure 2.**
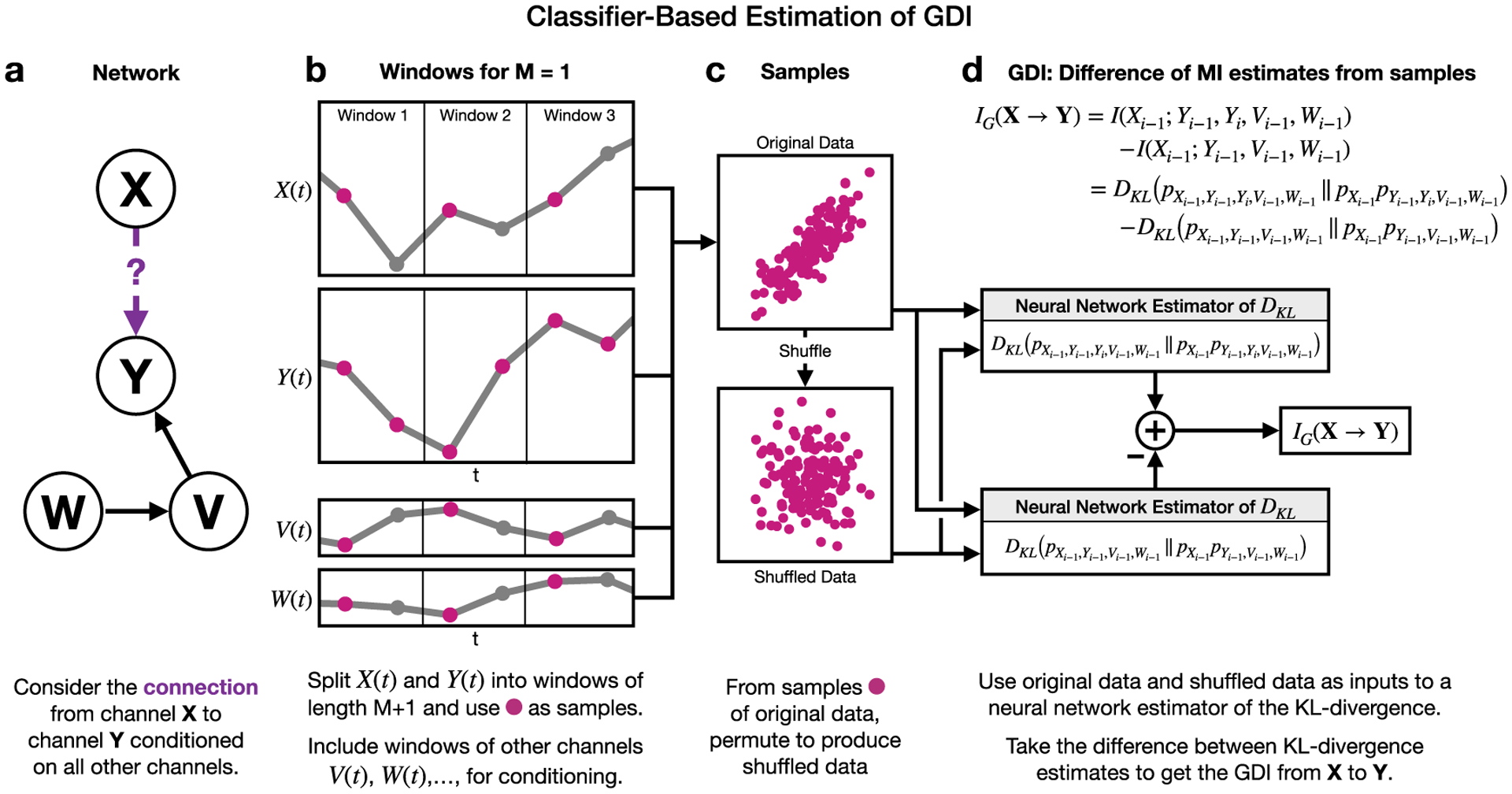
Overall process for estimating GDI between two nodes that relies on an MI estimation technique [14]. (a) Estimating GDI from **X** to **Y** includes conditioning on other nodes. For this visualization we specify two other nodes **V** and **W**. (b) First take windows of length *M* + 1 of nodes’ corresponding time series with *M* = 1 for this example. Gray points in windows are ignored because *I*_*G*_(**X** → **Y**) does not include values at time *i* except for *Y*_*i*_, which is because GDI is focused on causal rather than synchronous relationships. (c) Use values from windows as samples that capture joint statistics, and permute values to produce samples that capture independence. Permuted samples consist of windows from each time series that are no longer simultaneous, i.e. windows are effectively drawn independently from each time series in random orders. (d) Because GDI can be defined as the difference between two MI terms and therefore two KL-divergence terms, samples are used to estimate KL-divergences between the original data distributions and the shuffled data distributions. The extent to which a classifier can differentiate between the original and the shuffled data is quantified as MI, and the difference between the two MI terms provides an estimate of GDI.

Figure 2 illustrates how this classifier-based KL-divergence estimation approach can be applied to estimate the GDI between two nodes *X* and *Y* conditioned on two other nodes *W* and *V*. For the more general case, consider recordings from *R* channels where we want to estimate the GDI between channels *j* and *k*. The definition of CMI (4) allows this to be formulated as a difference of two MI terms:

(14) $$I\left(X^{\left(j\right)}\to X^{\left(k\right)}|X^{*}\right)=I(X_{i-M}^{\left(j\right)},\ldots ,X_{i-1}^{\left(j\right)};X_{i}^{\left(k\right)}|X_{i-M}^{\left(1\right)},\ldots ,X_{i-1}^{\left(1\right)},\ldots ,X_{i-M}^{\left(R\right)},\ldots ,X_{i-1}^{\left(R\right)})$$

(15) $$=I(X_{i-M}^{\left(j\right)},\ldots ,X_{i-1}^{\left(j\right)};X_{i}^{\left(k\right)},X_{i-M}^{\left(1\right)},\ldots ,X_{i-1}^{\left(1\right)},\ldots ,X_{i-M}^{\left(R\right)},\ldots ,X_{i-1}^{\left(R\right)})-I(X_{i-M}^{\left(j\right)},\ldots ,X_{i-1}^{\left(j\right)};X_{i-M}^{\left(1\right)},\ldots ,X_{i-1}^{\left(1\right)},\ldots ,X_{i-M}^{\left(R\right)},\ldots ,X_{i-1}^{\left(R\right)}),$$

where the second half of the *I* expressions on the right-hand side exclude $X_{i-M}^{\left(j\right)},\ldots ,X_{i-1}^{\left(j\right)}$. To estimate a given MI term in (15), non-overlapping windows of length *M* + 1 of each of the *R* channels are taken as shown in figure 2(b). The first *M* values of each window of a given channel *r* can be used as samples of the terms $X_{i-M}^{\left(r\right)},\ldots ,X_{i-1}^{\left(r\right)}$, while the last value of each window of channel *k* can be used as samples of the term $X_{i}^{\left(k\right)}$. All of these samples are effectively being drawn from a massive joint distribution *p*_*X*_ (13) which describes the relationships manifested in the data. However, in order to use (13) to estimate MI we also need samples drawn from *q*_*X*_, which represents the distribution where all variables are independent. An approach is to simply permute the samples of *p*_*X*_ [14], because this will simulate independent drawings (figure 2(c)). A portion of these samples for *p*_*X*_ and *q*_*X*_ can then be used to learn L via training a binary classifier to distinguish between samples from these two distributions. This allows the remainder of the samples to be plugged into (13) to provide a single MI estimate (figure 2(d)). The training and testing procedure is then repeated a number of times in a bootstrapped manner as outlined in the previous paragraph in order to provide an average MI estimate. By applying this overall KL-divergence approach to each MI term in (15), we arrive at the estimate of *I*_*G*_(**X**^(**j**)^ → **X**^(**k**)^).

### 2.4. Connection sign inference

Although information theoretic methods are powerful because of their generality, such methods do not indicate the positivity or negativity of a relationship. In the case of neural data, this means that DI alone cannot indicate whether a connection is excitatory or inhibitory, and therefore we need a sign inference method integrated into the GDI approach. Prior methods to infer the sign of a connection have included the use of cross-correlations [30] or a similar approach [1] where a presynaptic spike increasing the probability of a postsynaptic spike indicates an excitatory connection while a decrease would indicate inhibition. The chief issue that these pairwise methods fail to account for is the possibility of such inferences being contaminated by the influences of other nodes.

Consequently, we implement the partial correlation technique as a function of a time lag, which is similar to the approach of [31] and also similar to that of [32], which did not include a time lag. Although this is a linear method, we find that it is an effective approximation in practice. Similar to [33], we introduce the time-lagged correlation coefficient (TLC):

(16) $$\rho_{XY}(\tau )=\frac{\sum_{i}\left(x_{i}-\bar{x}\right)\left(y_{i-\tau }-\bar{y}\right)}{\sqrt{\sum_{i}{\left(x_{i}-\bar{x}\right)}^{2}\sum_{i}{\left(y_{i-\tau }-\bar{y}\right)}^{2}}},$$

where $\bar{x}$ and $\bar{y}$ denote the means of each time series while *τ* is the time lag between **X** and **Y**. This centering of the data performed by mean subtraction provides clear intuition for the TLC: if **X** and **Y** tend to be above (and below) their mean together then the TLC will be positive, while it will be negative if **X** tends to be above its mean while **Y** is below its mean and vice-versa. Identifying a relationship from **X** to **Y** as excitatory or inhibitory then reduces to simply taking the sign of the TLC where positive corresponds to excitatory and negative corresponds to inhibitory, which is similar to one step taken in [30]. Overall, this is basically a translation of another prior technique [1] that was focused on spike trains to now be used on continuously valued time series. We note that this translated approach alleviates the need of selecting a window width parameter required in the baselining approach [30].

However, none of these exclusively pairwise methods consider the influence of other nodes, and therefore one additional step must be taken. This final step is the introduction of the time-lagged partial correlation coefficient (TLPC) [31, 34]:

(17) $$\rho_{XY|W}(\tau )=\frac{\rho_{XY}(\tau )-\rho_{XW}(0)\rho_{WY}(\tau )}{\sqrt{1-\rho_{XW}^{2}(0)}\sqrt{1-\rho_{WY}^{2}(\tau )}},$$

which captures the TLC between **X** and **Y** adjusted for the activity of a third time series **W**. In considering more time series, {**X**^(1)^, **X**^(2)^, …, **X**^(**R**)^}, TLPC can in fact be further extended to condition on an arbitrary number of time series and we display a recursive definition of this with simplified notation for presentation purposes [31]:

(18) $$\rho_{jk|*}(\tau )=\frac{\rho_{jk|(*\setminus i)}(\tau )-\rho_{ji|(*\setminus i)}(0)\rho_{ik|(*\setminus i)}(\tau )}{\sqrt{1-\rho_{ji|(*\setminus i)}^{2}(0)}\sqrt{1-\rho_{ik|(*\setminus i)}^{2}(\tau )}},$$

where subscripts **i**, **j**, **k**, and * denote **X**^(**i**)^, **X**^(**j**)^, **X**^(**k**)^, and **X**^(^*^)^, and because **X**^(^*^)^ = {**X**^(1)^, **X**^(2)^, …, **X**^(**R**)^} \ { **X**^(**j**)^, **X**^(**k**)^}, the subscript (* \ **i**) denotes **X**^(^*^)^\ **X**^(**i**)^. We then use TLPC to infer the nature of a connection in a manner reminiscent of [30]:

(19) $$S^{\left(j,k\right)}=sgn\left(\rho_{jk|*}\left(\tau^{*}\right)\right),\;\tau^{*}={\text{arg max}}_{\tau \in \mathbb{Z}-}\left[\left|\rho_{jk|*}(\tau )\right|\right],$$

where *sgn* is the sign function and *S*^(*j*,*k*)^ will either be −1 (inhibition) or +1 (excitation). To summarize, one first finds the delay τ* that is less than zero and that produces the maximum absolute value of the partial correlation between **X**^(**j**)^ and **X**^(**k**)^ conditioned on all other time series. Then, the sign of the partial correlation at that delay is taken to be the sign of the connection from **X**^(**j**)^ to **X**^(**k**)^, and this sign is applied to GDI estimates. The edge weights in the resulting DI graph are each quantified by the signed GDI as *w*^(*j*,*k*)^ = *S*^(*j*,*k*)^ × *I*_*G*_(**X**^(**j**)^ → **X**^**(k**)^) as per (11).

**Figure 3.**
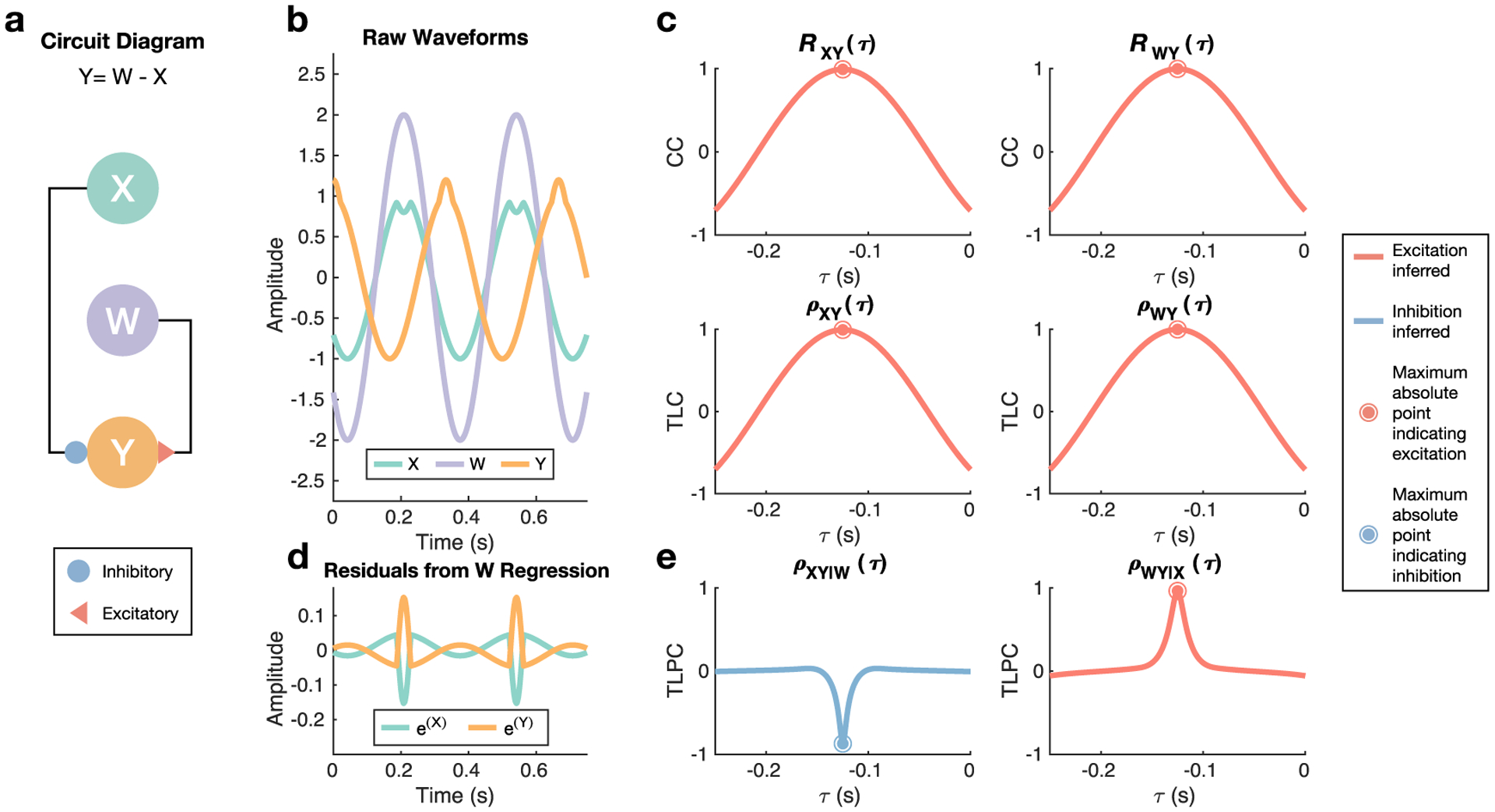
Continuously-valued example demonstrating the ability of graph theoretic methods to correctly differentiate excitatory and inhibitory connections. (a) A simple three-node system was analyzed, where **X** inhibits **Y** and **W** excites **Y**, which can be summarized as *Y*(*t*) = *W*(*t* − 0.125) − *X*(*t* − 0.125). (b) Snapshot of the sinusoidal waveforms generated. The slight dip around every peak of **X** combined with the typical peak of **W** produces the delayed but emphasized peak of **Y** 0.125 s later. If **X** did not exhibit this dip, it would be impossible to identify **X** as inhibitory because its activity would be fully masked by the excitatory effect of **W**. (c) Using the peak of the normalized cross-correlation (CC) *R*_**XY**_ or the time lagged correlation (TLC) *ρ*_**XY**_ results in incorrect identification of the connection from **X** to **Y** as excitatory. However, both methods do result in correct identification of the connection from **W** to **Y** as excitatory. (d) In order to perform partial inference, which accounts for the activity of other nodes in pairwise analysis, the residuals resulting from regressing **X** and **Y** against **W** (pictured) as well as from regressing **W** and **Y** against **X** are obtained. (e) Time lagged partial correlation (TLPC), i.e. the correlation coefficient between the residuals at different time lags, correctly identifies both the inhibitory and excitatory connections by accounting for other nodes’ activity.

**Figure 4.**
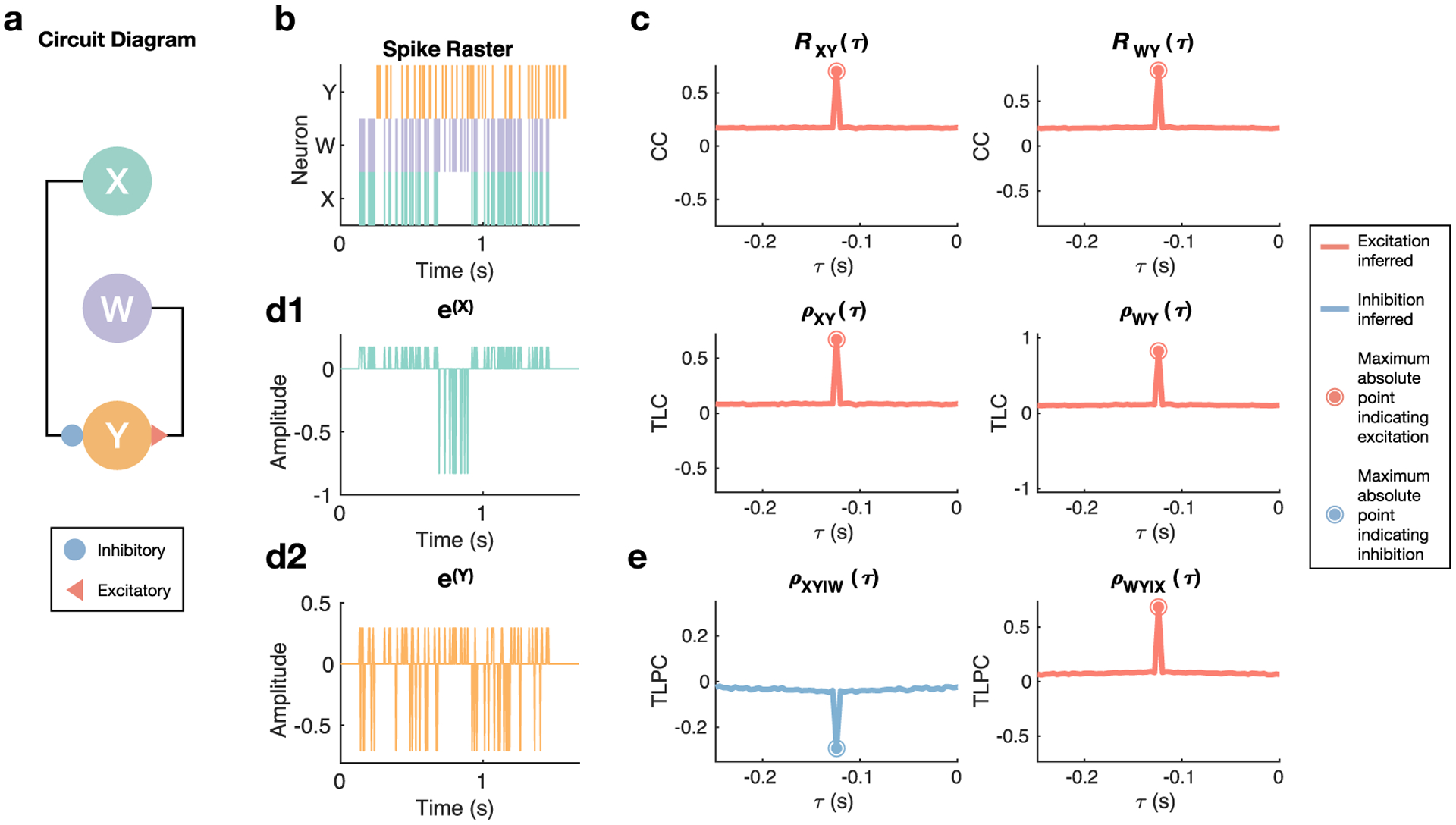
Discretely-valued example demonstrating the ability of graph theoretic methods to correctly differentiate excitatory and inhibitory connections. (a) A simple three-node system was analyzed, where **X** inhibits **Y** and **W** excites **Y**, both with a delay of approximately 0.125 s. (b) Snapshot of the spikes generated. The intermittent dropout of **X** allows for enhanced excitation of **Y** by **W**. (c) Both the normalized cross-correlation (CC) *R*_**XY**_ and time lagged correlation (TLC) *ρ*_**XY**_ fail to identify the connection from **X** to **Y** as inhibitory via use of peak values. However, both methods do correctly identify the connection from **W** to **Y** as excitatory. (d) In order to perform partial inference, which accounts for the activity of other nodes in pairwise analysis, the residuals resulting from regressing **X** (d1) and **Y** (d2) against **W** as well as from regressing **W** and **Y** against **X** are obtained. (e) Time lagged partial correlation (TLPC), i.e. the correlation coefficient between the residuals at different time lags, correctly identifies both the inhibitory and excitatory connections by accounting for other nodes’ activity.

To demonstrate the advantage of the conditional approach to sign inference, we use an example for both the continuously-valued case (figure 3) and the discretely-valued case (figure 4). Although the simulated networks of the results will involve 11 and 13 node systems where many nodes are conditioned, we consider a three-node system here for demonstration purposes. Formally, node **X** inhibits **Y** and node **W** excites **Y**, both with a delay of 0.125 s in their effects (figure 3(a)). The key feature of this simulation is that although **X** inhibits **Y**, this effect is almost completely masked by the excitatory effect of **W** on **Y** (figure 3(b)). There is a moment in each cycle of the system where **X** experiences a dip in its activity, which is reflected later as a momentary increase in the activity of **Y** because it is less inhibited. If this dip did not exist, then it would be impossible to identify **X** as inhibitory since the excitatory effect of **W** would fully mask the influence of **X**. Notably, neither the normalized CC *R*_**XY**_ nor TLC *ρ*_**XY**_ notice the inhibitory effect of **X** on **Y**, instead clearly labeling the effect as excitatory as evidenced by both methods detecting a (positive) peak (figure 3(c)). The TLPC method overcomes this issue by regressing out other time series before considering the correlation between two particular time series, which is an equivalent approach to computing TLPC (18) [35]. In particular, one would use simple linear regression where $x_{i}=\beta^{\left(w,x\right)}w_{i}+\epsilon_{i}^{\left(x\right)}$ and $y_{i-\tau }=\beta^{\left(w,y\right)}w_{i}+\epsilon_{i}^{\left(y\right)}$ to estimate the residuals $\epsilon_{i}^{\left(x\right)}$ and $\epsilon_{i}^{\left(y\right)}$, which are shown in figure 3(d), and the TLPC would then be the correlation coefficient between these two residuals. We note that the residual for **Y** is shown with a delay *τ* corresponding to −0.125 s in order to highlight the inhibitory effect of **X** on **Y** that is clear after regressing out **W**. This inhibitory effect is correctly identified by TLPC (figure 3(e)), i.e. the correlation coefficient between these residuals, where a clear negative trough is detected in contrast to the positive peaks seen in the normalized CC and TLC resulting from not adjusting for **W**. We conclude by noting that all three methods do identify the excitatory effect from **W** to **Y** correctly, which is sensible because its effect is not masked by **X**.

Figure 4 closely follows the explanation presented in the prior paragraph, however discretely-valued data reminiscent of binned spike times replaces the continuously-valued simulation used in figure 3. The key finding is the same between these figures, namely that TLPC is able to identify an inhibitory connection from **X** to **Y** that would otherwise remain hidden when using CC or TLC because of the masking effect of the excitatory connection from **W** to **Y**. Using this partial correlation approach on discretely-valued data in the form of binned spike times means that *x*_*i*_ and *y*_*i*−*τ*_ will take on 0 (no spike) or 1 (spike).

## 3. Results

We begin by illustrating how the GDI technique scales with sample size, dimensionality, and bootstrapping. We then apply GDI to a variety of models where many nodes are conditioned in order to highlight the importance of a graphical method in network analysis. Initially, we apply GDI to continuously valued data analogous to voltage time series in a Gaussian simulation and a nonlinear simulation. Next, we apply GDI to binned spike times generated by networks consisting of conductance-based realistic spiking neurons, where the first network is arbitrary and the second network is a model of a central pattern generator (CPG) circuit in *Aplysia*. The general finding of these results is that GDI’s conditioning performance provides significant reductions in indirect connectivity, producing more accurate connectivity analyses.

### 3.1. Scaling performance

**Figure 5.**
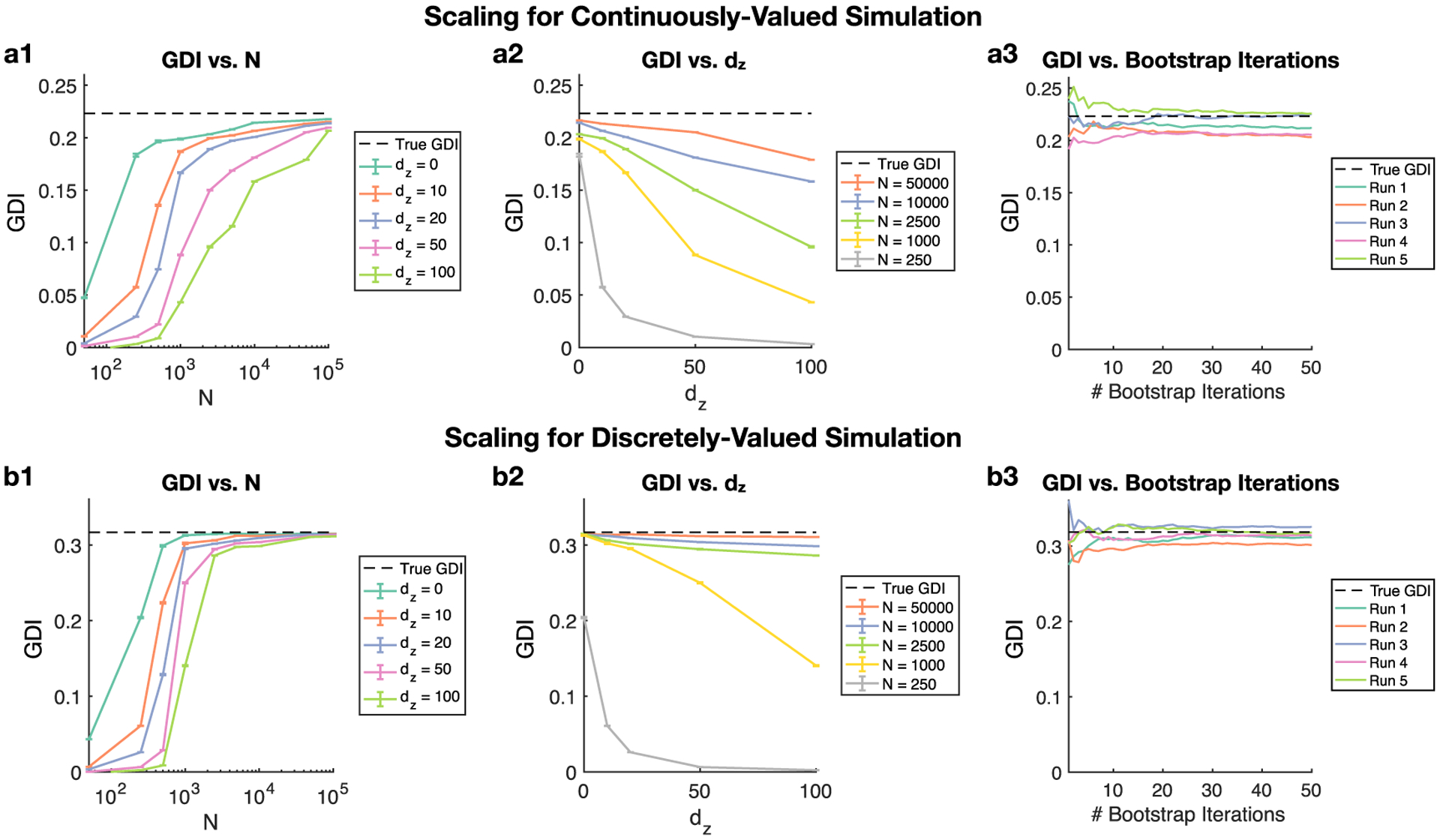
Scaling plots. (a) A Gaussian simulation was implemented where *ρ* = 0.6. (b) A binary symmetric channel simulation was implemented where *X*_*i*−1_~Bernoulli(0.3), Pr[*Y*_*i*_ = *X*_*i*−1_] = 0.9, and *I*(**X**→**Y**) is known analytically. (a1,b1) Analysis of how GDI scales with number of samples *N*. Error bars indicate mean and variance of GDI estimates across 50 independent simulations with 10 bootstrap iterations used per estimate. *d*_*z*_ indicates number of dimensions being conditioned, which in the top row corresponds to independent unit Gaussian random variables and in the bottom row corresponds to discrete uniform random variables (0 or 1). *d*_*z*_ is therefore proportional to the number of nodes being conditioned, and accordingly indicates how GDI scales with network size. (a2,b2) Analysis of how GDI scales with number of dimensions being conditioned for given sample sizes *N*. (a3,b3) Analysis of how GDI estimates converge as number of bootstrap iterations increases. Each line is the cumulative average GDI estimate over bootstrap iterations for an independent simulation with *d*_*z*_ = 0 and *N* = 2500 samples.

Figure 5 illustrates how the GDI estimator performs with regards to the sample size *N* used for estimation, the number of dimensions *d*_*z*_ being conditioned, and the number of bootstrap iterations used to train and test the classifiers. Because *d*_*z*_ is proportional to the number of nodes being conditioned, it provides a measure of how GDI scales with network size. We consider both continuously-valued (figure 5(a)) and discretely-valued (figure 5(b)) examples. For the continuously-valued simulation, we implement a bivariate Gaussian model where $\left(X_{i-1},Y_{i}\right)\sim N\left(\left[0\;0\right],\left[1\;\rho ;\rho \;1\right]\right)$ with *ρ* = 0.6. This means that the DI between **X** and **Y** is simply a function of the covariance, i.e. $I(X\to Y)=-\frac{1}{2}\text{log}\left(1-\rho^{2}\right)$ [18]. For the discretely-valued simulation, we implement a binary symmetric channel model where $\text{Pr}\left[Y_{i}={\bar{X}}_{i-1}\right]=p$ and Pr[*Y*_*i*_ = *X*_*i*−1_] = 1 − *p*, where ${\bar{X}}_{i-1}$ denotes the logical complement of *X*_*i*_ − 1 and *p* = 0.1 which is the probability of a bit flip. We further note that *X*_*i*−1_~Bernoulli(0.3) which means that the analytic GDI can be computed exactly by the discrete version of (2).

When we consider the effects of the number of dimensions *d*_*z*_ being conditioned, we use two different models. For the continuously-valued scenario, independent unit Gaussian random variables are being conditioned. For example, *d*_*z*_ = 10 means that the DI estimate from **X** to **Y** is being conditioned on 10 Gaussian variables. For the discretely-valued scenario, independent discrete uniform random variables defined on the set {0, 1} are being conditioned. Increasing *d*_*z*_ provides a sense of how GDI scales with dimensionality.

The first scaling analysis compares GDI estimates with the number of samples *N* (figures 5(a1) and (b1)), where each point is the average estimate across 50 independent simulations with 10 bootstrap iterations used per estimate. Each trace corresponds to a particular dimensionality *d*_*z*_ of what is being conditioned in the GDI estimate. As expected, estimates across *d*_*z*_ values improve with the number of samples. Notably, in the continuous-valued case the estimates with *N* = 2500 samples where *d*_*z*_ = {0, 10, 20} differ by little despite large differences in dimensionality. Moreover, the performance of the GDI estimator is better for the discretely-valued simulation for *N* = 2500 samples, where reliable estimates for even *d*_*z*_ = 100 are seen. In general, the variance of estimates is very low. We finally point out that *d*_*z*_ = 0 corresponds to regular, pairwise DI, which displays how this technique performs as a standard DI estimator.

The second scaling analysis compares GDI estimates with the dimensionality *d*_*z*_ of what is being conditioned for given sample sizes (figures 5(a2) and (b2)), which is an alternative way to look at the analysis considered in figures 5(a1) and (b1). As expected, estimates decay relative to the true value with increasing *d*_*z*_, no matter what sample size is used. However, for the majority of considered sample sizes the decay is quite linear, which is notable given the dimensionalities being considered.

The third scaling analysis compares GDI estimates with the number of bootstrap iterations used to train and test the classifier that is at the core of GDI estimation (figures 5(a3) and (b3)). A GDI estimate is the average of bootstrapped estimates, which each come from randomly splitting the data into training and testing sets for the classifier used in GDI estimation. Therefore, we plot the cumulative average GDI estimate over bootstrap iterations for given runs of the simulations (figures 5(a3) and (b3)) with *d*_*z*_ = 0 and *N* = 2500 samples. These plots show that GDI estimates converge when given enough bootstrap iterations. For the remaining results, we use at minimum 100 bootstrap iterations to help with convergence of our GDI estimates except for the final CPG result where a different scheme is used. In general, we recommend consideration of the level of variability that can occur in GDI estimates depending on the number of bootstrap iterations used, particularly since the analyses for figures 5(a3) and (b3) are specific to a given model and parameters for that model.

Overall, scaling analysis reveals that GDI can condition on many nodes without requiring an inordinate number of samples. This scaling performance enables the following network-level analyses.

### 3.2. Gaussian network

**Figure 6.**
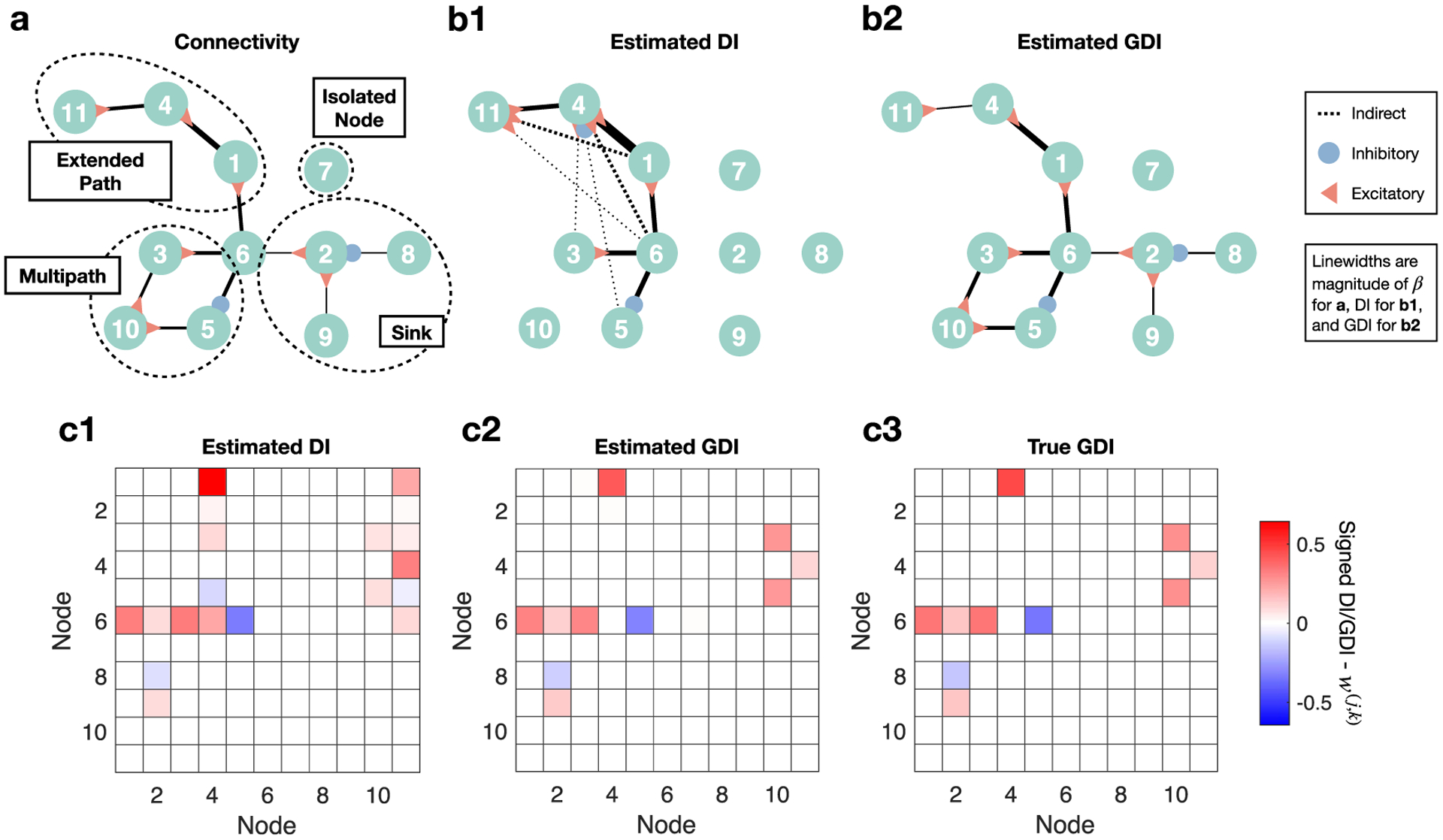
Analysis of signed GDI performance on Gaussian network. (a) The network structure incorporates four motifs: an extended path where there is a trail of connections from node 6 to 11, a sink where 3 other nodes influence node 2, a multipath connection from node 6 to 10, and an isolated node. Each connection indicates that the most recent past value of the causal node influences the current value of the node with a circle (inhibited) or triangle (excited). (b1) Considering the 10 highest edges, signed non-GDI, i.e. DI without conditioning on other nodes, detects a number of indirect connections and therefore misses a number of direct connections. (b2) Ten highest signed GDI values are the true direct connections and do not include any indirect connections, which is achieved by its conditioning ability. (c) Comparing all estimated DI (c1), estimated GDI (c2), and analytic GDI (c3) values. DI misidentifies many false connections (c1), while GDI correctly recovers the graph (c2). Similarity between estimated GDI (c2) and analytic GDI (c3) highlights estimator accuracy.

We begin our first of two analyses analogous to studying voltages over time by considering a simulation of continuously valued time series where the analytic GDI is known. Knowledge of the analytic GDI enables the quantification of our estimator’s performance. Formally, consider *R* random processes {**X**^(1)^, **X**^(2)^, …, **X**^(**R**)^} where the *k*th process or node at time *i* follows the form $x_{i}^{\left(k\right)}=\sum_{j=1}^{R}\beta^{\left(j,k\right)}x_{i-1}^{\left(j\right)}+\alpha^{\left(k\right)}z_{i}^{\left(k\right)}$ where $z_{i}^{\left(k\right)}\sim N(0,1)$ and $\sum_{j=1}^{R}\beta^{\left(j,k\right)}+\alpha^{\left(k\right)}=1$. Simply put, each time series is a linear combination of a noise term and past values of other time series, which is inspired by a prior model for two nodes [3]. A graphical representation of a constructed version of this network is illustrated in figure 6(a). In this representation the presence of an edge from a given node *j* to node *k* indicates that *|β*^(*j*,*k*)^*|* > 0, where the linewidth is proportional to *|β*^(*j*,*k*)^*|* and the sign of *β*^(*j*,*k*)^ is indicated by a triangle (excitatory) or by a circle (inhibitory). The network contains four features: an extended path consisting of a series of connected nodes, a sink consisting of a single node influenced by multiple nodes simultaneously, a multipath connection from node 6 to node 10, and an isolated node. DI and GDI estimates from the simulated data of this network use a memory parameter of *M* = 3 past samples because the maximum lag within the network is 3 because of the extended path from node 6 to 11. We further note that 1e5 samples were used, and that 100 bootstrap iterations were used for splitting data into training and testing sets for the classifier used in GDI estimation.

If we only consider pairwise estimates of the signed DI between nodes (figures 6(b1) and (c1)), then a number of false connections are detected because there is no conditioning done on other nodes that may causally explain such links. Because it is known that ten true connections exist, it is interesting to analyze the ten DI inferences with the highest absolute values (figures 6(b1)). The extended path 6 → 1 → 4 → 11 is massively distorted by the pairwise DI analysis. Although pairwise DI detects the direct connections along the path, it also detects 5 different false indirect connections. Nodes 3 and 5 of the multipath structure become falsely associated with node 4 as part of the extended path, representing 2 of these false connections. Overall, these results can be connected with the data processing inequality (DPI), a well known concept in information theory which states that for a Markov chain *X* → *Y* → *Z* the following inequality must hold: *I*(*X*; *Z*) ⩽ *I*(*X*; *Y*) [18]. This extends naturally to DI and is observed in figure 6(b1) such as when *I*(**X**^(6)^ → **X**^(4)^) < *I*(**X**^(6)^ → **X**^(1)^). Notably, the true direct connections from nodes 3 and 5 to node 10 as well as those from nodes 6, 8, and 9 to node 2 all have pairwise DI estimates below those of the displayed indirect connections. Because some indirect connections have greater DI estimates than those of direct connections, we cannot simply remove the indirect connections via thresholding and preserve all of the direct connections with pairwise DI alone. Considering all of the inferred values beyond the ten highest reveals that DI detected additional false connections (figure 6(c1)). Finally, we note that for the connections that pairwise DI correctly identifies, the TLC infers the correct sign values. For the signed GDI analysis, we implement the partial version of this (TLPC).

In contrast to the errors made by signed pairwise DI, the ten highest signed GDI (figures 6(b2) and (c2)) values accurately recover the true connectivity structure without identifying any indirect connections, and the use of TLPC for sign inference yields the correct connection signs *S*^(*j*,*k*)^ as well. In particular, unlike pairwise DI the extended path structure is fully recovered by GDI because of its ability to condition on the activity of other nodes. Furthermore, GDI analysis removes the indirect connections that pairwise DI had incorrectly identified from nodes 3 and 5 to node 4. In summary, GDI’s conditioning ability has allowed us to threshold all of the indirect connections and preserve all of the direct connections, which we were unable to do with pairwise DI alone. Furthermore, all other GDI values apart from the ten true connections identified are negligible (figure 6(c2)).

Apart from structural accuracy, it is important to comment on the differences in the magnitude of edge weights between DI and GDI. The comparison between GDI and pairwise DI is similar to how partial correlations capture the *unique* covariation between two variables by controlling for other variables while correlations capture all covariation. For example, GDI (figure 6(c2)) in the sink structure is greater than pairwise DI (figure 6(c1)) because by conditioning the analysis of a single connection on the other 3 connections, which means that we remove the influence of the other 3 nodes on node 2, the influence of the single connection will appear relatively larger than inference without such removal. Later analytical analysis will mathematically clarify this point, and this same effect is realized for the connections from nodes 3 and 5 to node 10. The connections from node 6 to nodes 1, 3, and 5 are unaffected by conditioning because no other past activity would help explain the target nodes’ activity, resulting in DI and GDI being identical for these connections. Finally, we consider differences between DI and GDI for the last two connections of the extended path. GDI from node 1 to 4 is lower than DI because activity of node 6 is controlled for (removed), meaning GDI solely captures activity unique to node 1 that is causally expressed in node 4. This effect is seen to an even greater extent in the connection from node 4 to 11 where not only is the activity of node 6 controlled for but also that of node 1, resulting in GDI being significantly lower than DI.

Furthermore, despite the estimation of GDI being a 34-dimensional problem as one must consider the past 3 values for each of the 11 nodes along with the current value of the target node, our estimates are very accurate when compared with the true analytical GDI values (figures 6(c2) and (c3)) which are derived in the following paragraph. We emphasize that the values in the heatmap are not thresholded. In other words, the conditioning ability of GDI reduced estimates of indirect connections to nearly zero, as no indirect connections have a high enough value to visibly register on the heatmap (figure 6(c2)). We do note that there is the slight presence of estimation bias where estimated values tend to be lower than the true values, but the heatmaps make it clear that this bias is rather minor.

The analytical GDI values for this network can be determined by considering an analytical solution to a simpler problem from prior work [3]. Consider the scenario where only one node *j* influences one node *k*, i.e. $x_{i}^{\left(k\right)}=\beta^{\left(j,k\right)}x_{i-1}^{\left(j\right)}+\alpha^{\left(k\right)}z_{i}^{\left(k\right)}$. Both $x_{i-1}^{\left(j\right)}$ and $z_{i}^{\left(k\right)}$ follow a standard Gaussian distribution N(0,1), which means that the variance of $\beta^{\left(j,k\right)}x_{i-1}^{\left(j\right)}$ will be ${\left(\beta^{\left(j,k\right)}\right)}^{2}$ and the variance of $\alpha^{\left(k\right)}z_{i}^{\left(k\right)}$ will be ${\left(\alpha^{\left(k\right)}\right)}^{2}$ The DI from node *j* to node *k* is then simply [3]:

(20) $$I\left(X^{\left(j\right)}\to X^{\left(k\right)}\right)=\frac{1}{2}\text{log }\left(1+\frac{{\left(\beta^{\left(j,k\right)}\right)}^{2}}{{\left(\alpha^{\left(k\right)}\right)}^{2}}\right),$$

which is the DI for the connections from nodes 1 to 4 and nodes 4 to 11, and which will also equal both the DI and GDI for the connections from node 6 to nodes 1, 3, and 5. For a connection in the sink structure or in the multipath structure from nodes 3 or 5 to 10, other connections will appear to increase the noise variance when estimating DI. This means that the DI from node *j* to *k* would be similar to (20) but with an increase in the denominator (noise) term:

(21) $$I\left(X^{\left(j\right)}\to X^{\left(k\right)}\right)=\frac{1}{2}\text{log }\left(1+\frac{{\left(\beta^{\left(j,k\right)}\right)}^{2}}{{\left(\alpha^{\left(k\right)}\right)}^{2}+\sum_{l}{\left(\beta^{\left(l,k\right)}\right)}^{2}}\right),$$

where *l* ∈ {1, 2, …, *R*} \ {*j*, *k*}. For connections in the sink structure GDI will condition and remove the influence of the other nodes meaning that their appearance as noise will disappear and GDI is simply (20). This results in GDI being greater than DI for the sink connections because what is effectively the signal-to-noise ratio (SNR) term is inherently higher in the GDI equation than in the DI equation. The remaining terms *I*_*G*_(**X**^(1)^ → **X**^(4)^), *I*_*G*_(**X**^(4)^ → **X**^(11)^), *I*_*G*_(**X**^(3)^ → **X**^(10)^), and *I*_*G*_(**X**^(5)^ → **X**^(10)^) can be found to follow the form of (20) where the fraction inside log additionally includes (*α*^(*j*)^)^2^ and becomes (*β*^(*j*,*k*)^)^2^(*α*^(*j*)^)^2^/(*α*^(*k*)^)^2^.

### 3.3. Nonlinear network

**Figure 7.**
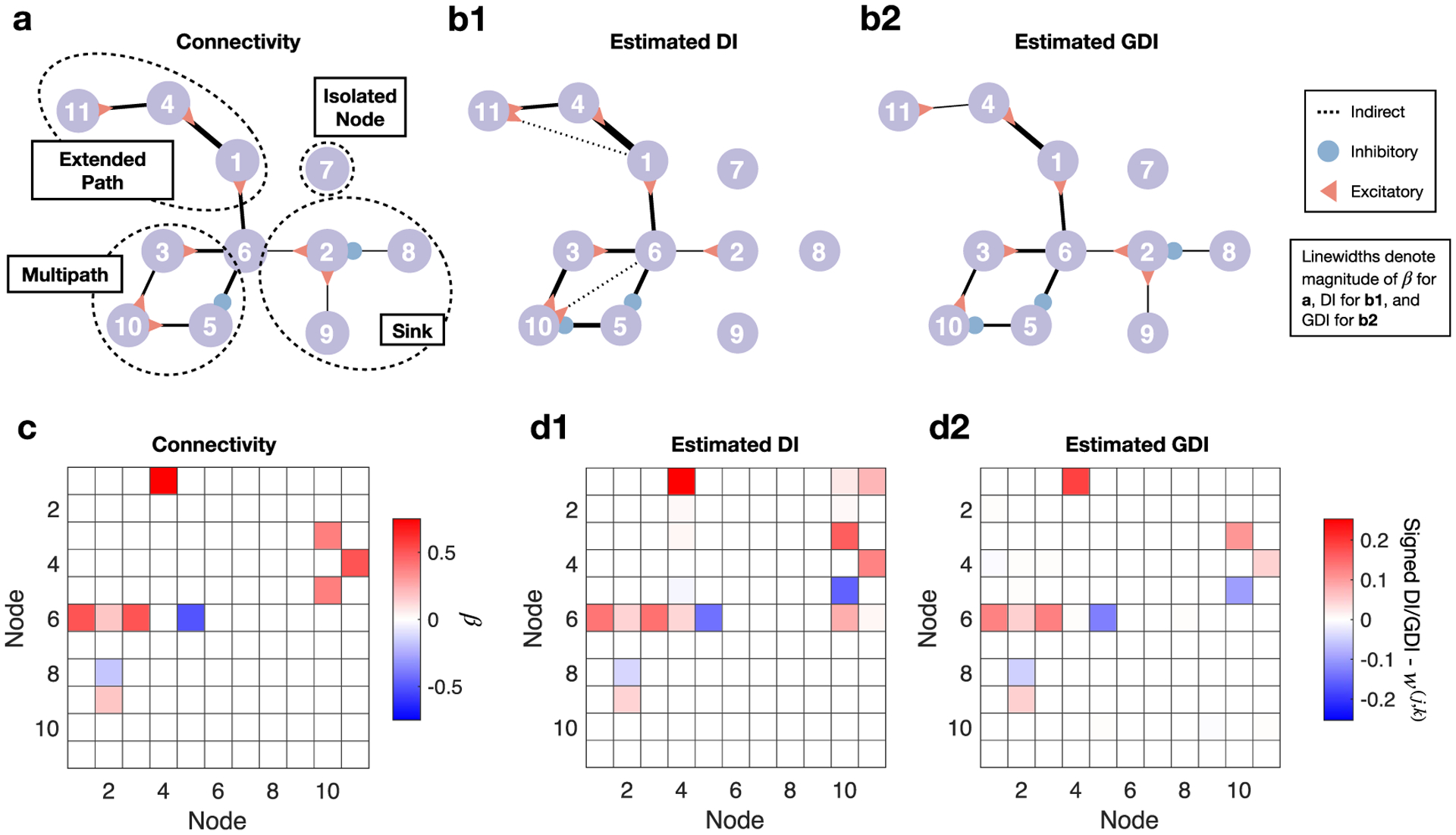
Analysis of GDI performance on non-Gaussian and nonlinear network. (a) The network structure is the same as that of the prior figure, incorporating four motifs: an extended path where there is a trail of connections from nodes 6 to 11, a sink where 3 other nodes influence node 2, a multipath connection from nodes 6 to 10, and an isolated node. Each connection indicates that the most recent past value of the causal node influences the current value of the node with a circle (inhibited) or triangle (excited). (b1) Considering the ten greatest edges, non-GDI misidentifies two indirect connections and therefore misses two direct connections. (b2) Considering the ten greatest edges, GDI correctly identifies the true direct connectivity structure and does not identify any indirect connections, which is achieved by its conditioning ability. (c) Heatmap of true connectivity structure. (d) Comparing all values of estimated DI (d1), which mistakenly identifies indirect connections, and GDI (d2), which correctly identifies the direct connections and reduces the indirect connections to negligible values.

It is unlikely that voltage recordings of neural activity are completely Gaussian and have completely linear interrelationships. As a demonstration of the generality of GDI, we consider a simulation (figure 7(a)) that has the same connectivity structure as the prior subsection but contains non-Gaussian activity and nonlinear interactions. More specifically, although the *β* and *α* values are the same as the previous values, two changes were implemented.

First, the values at any given time of source nodes 6, 8, and 9, as well as the isolated node 7, are drawn from continuous uniform random variables defined on the range (0, 1). Even though the additive noise terms (*z*_*i*_) in the other nodes remain Gaussian random variables, the introduction of uniform random variables at the source nodes causes non-Gaussian activity to be propagated throughout the entire network.

Second, we introduce nonlinearity into the network by replacing the linear relationship between past values of source nodes and current values of target nodes with a square relationship, which is a causal network version of a previously considered model [3]. Mathematically, activity of target nodes can now be modeled as $x_{i}^{\left(k\right)}=\sum_{j=1}^{R}\beta^{\left(j,k\right)}{\left(x_{i-1}^{\left(j\right)}\right)}^{2}+\alpha^{\left(k\right)}z_{i}^{\left(k\right)}$. Again, the *z*_*i*_ terms in target nodes are still drawn from Gaussian random variables, however the variance is now 0.25 rather than 1 in order to counter the low ${\left(x_{i-1}^{\left(j\right)}\right)}^{2}$ values resulting from the square of a number less than 1.

Just as with the prior subsection (figure 6), DI and GDI estimates from the simulated data of this network use a memory parameter of *M* = 3 past samples, 1e5 samples of the simulation were generated, and 100 bootstrap iterations were used for the classifiers. We note that unlike the linear Gaussian example of the prior subsection, no analytic solutions are known for the DI and GDI values of this network, however we are still able to compare our results with what we know to be the true connectivity structure displayed in figure 7(a).

If we only consider the ten greatest pairwise estimates of the signed DI between nodes (figure 7(b1)), then two indirect connections are included because of unawareness of the activity of other nodes that may causally explain such links. One of these is from node 1 to node 11, distorting the true nature of the extended path motif. The other included indirect connection is within the multipath structure, being from node 6 to node 10 which similarly distorts the true structure. As in the prior subsection, these results can be connected with the DPI [18] when we observe in figure 7(b1) that *I*(**X**^(1)^ → **X**^(11)^) < *I*(**X**^(1)^ → **X**^(4)^) and *I*(**X**^(6)^ → **X**^(10)^) < *I*(**X**^(6)^ → **X**^(3)^). Notably, the true direct connections from nodes 8 and 9 to node 2 both have pairwise DI estimates below those of the displayed indirect connections. As before, this means that with pairwise DI alone we cannot simply threshold the indirect connections and preserve all of the direct connections. Furthermore, considering all pairwise DI estimates (figure 7(d1)) in comparison with a heatmap of the true connectivity structure (figure 7(c)) reveals that non-GDI misidentified other indirect connections as well. Finally, we note that for the connections that pairwise DI correctly identifies, the TLC infers the correct sign values except for the connection from node 5 to node 10. This sign inference error shows that although our linear approach to sign inference performs well, it does have limitations in addressing more complex phenomena and this applies not only to TLC but also to TLPC.

In contrast to the errors made by signed pairwise DI, signed GDI (figures 7(b2) and (d2)) recovers the true connectivity structure without identifying any indirect connections. Importantly, the ten greatest values of signed GDI (figure 7(b2)) are the true direct connections, and all other GDI estimates are negligible (figure 7(d2)). With the exception of the connection from node 5 to node 10, the use of TLPC for sign inference yields the correct connection signs *S*^(*j*,*k*)^ as well. Unlike pairwise DI, the extended path structure is fully recovered by GDI in a fashion similar to that of the prior example. Furthermore, the indirect connection from node 6 to node 10 is removed and the connections from nodes 8 and 9 to node 2 are recovered. In essence, GDI’s conditioning performance has enabled us to threshold all of the indirect connections and retain all of the direct connections, which was not possible with pairwise DI alone.

### 3.4. Network with conductance-based spiking neurons

**Figure 8.**
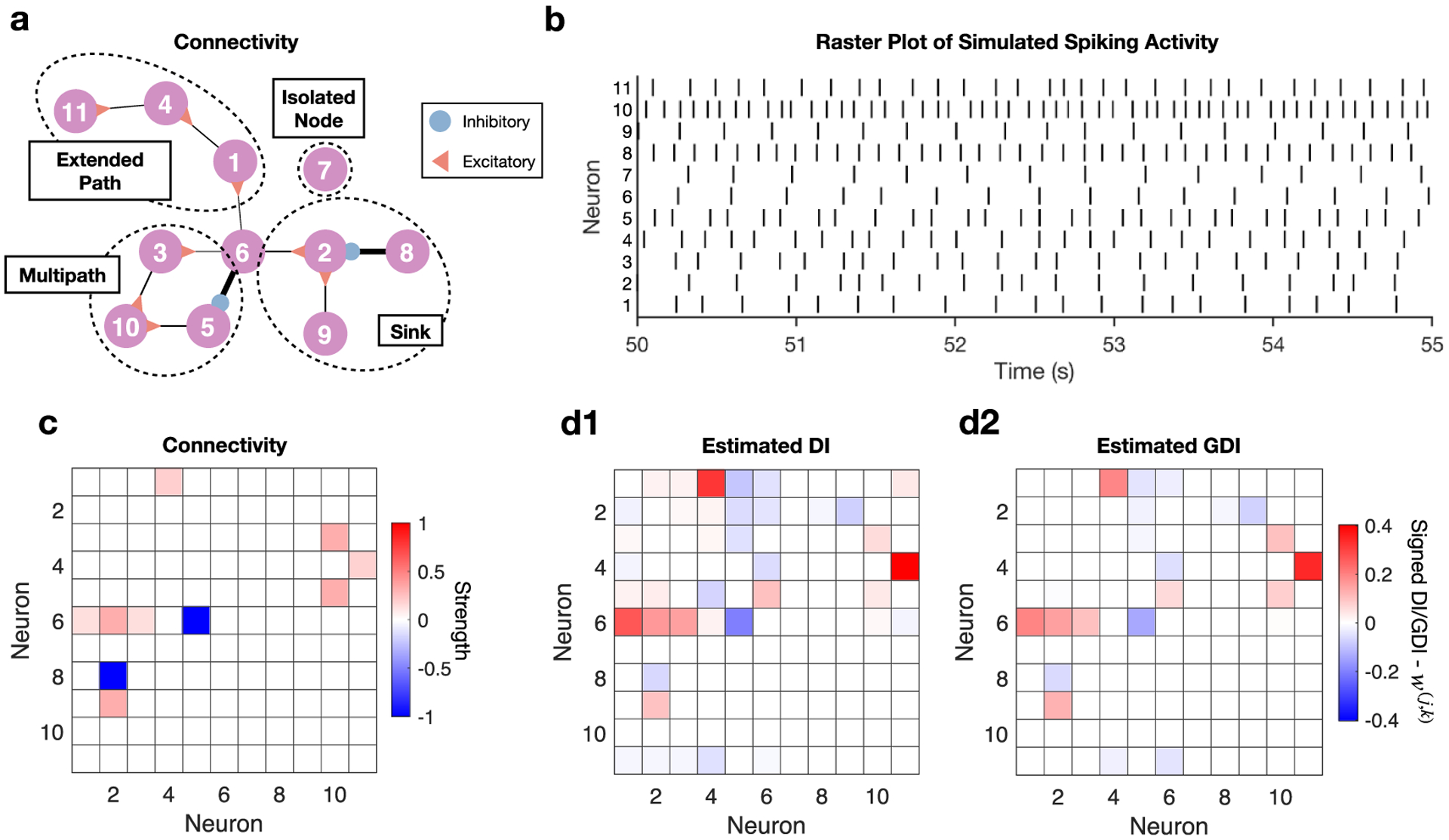
Analysis of signed GDI performance on network of conductance-based spiking neurons with same edge structure as prior two examples, however weights have been modified. (a) Structure of direct connectivity between neurons. Linewidths proportional to synaptic strength. (b) Raster plot of simulated spike times. (c) Heatmap of direct connectivity between neurons. Color intensity proportional to synaptic strength. (d1) DI detects the direct connections but also includes many false positives. (d2) GDI analysis minimizes indirect connections by conditioning pairwise analyses on other simulated neurons’ activity, better characterizing the true connectivity structure.

We next examined the ability of a graphical method to exclude indirect connections by applying GDI to discretely-valued data such as binned spike times from physiological network models. In particular, we examined two conductance-based neuronal models implemented via SNNAP (Simulator for Neural Networks and Action Potentials) [36–39]. Figure 8(a) displays the network structure of the first model, which consists of both excitatory and inhibitory connections in the same configuration as the two previous samples, except the weights have been altered. Linewidths in figure 8(a) are proportional to synaptic strengths. The files Zenodo (https://doi.org/10.5281/zenodo.4207536) for this network simulation, which contain the relevant parameters, are included at the link provided in the Data availability section. A snapshot of the raster plot resulting from a simulation of this network is shown in figure 8(b). The simulation was run for 300 s, a bin with of 35 ms was used for binning the spike times, and a memory parameter of *M* = 3 was used for DI and GDI estimation with the number of bootstrap iterations set to 500.

In addition, for the discretely-valued analyses, a normalization factor from prior work [1] has been employed that changes the scale of DI so that it ranges from 0 to 1, because without the factor DI is non-negative but otherwise unbounded. An added benefit of this factor is that, relative to the target node’s information, incoming information is normalized [1]. We use this factor for GDI as well so that values between DI and GDI can be compared. Furthermore, because a lower sample size than the prior two examples is being considered, we have GDI only condition on other nodes that also have significant DI equal to or greater than 0.01 with the target node, a threshold based on prior work [1] that will improve accuracy by keeping dimensionality low. We note that this will cause dimensionalities to be different between GDI estimates, which means the bias and variance of such estimates will differ. However, this is preferable to the alternative strategy of unnecessarily conditioning on every other node which will introduce greater bias and variance in general.

With figure 8(c) serving as a reference for the true connectivity structure, figure 8(d1) and figure 8(d2) display the connectivities extracted via simple pairwise DI analysis and via GDI, respectively. Non-GDI does identify the true direct connections, however it also misidentifies many false positives (figure 8(d1)). By contrast, GDI minimizes the vast majority of indirect connections while preserving the direct connections (figure 8(d2)). For example, a number of false connections that DI (figure 8(d1)) misidentified in roughly the top left quadrant have been reduced or eliminated by GDI (figure 8(d2)). Notably, the false connection from neuron 1 to 11 misidentified by DI (figure 8(d1)) was completely eliminated by GDI (figure 8(d2)). Overall, the conditioning ability of GDI has adjusted edge weights so that more of the connections are true direct connections than in comparison to the weights found by simply pairwise DI analysis.

### 3.5. Aplysia central pattern generator (CPG) circuit

**Figure 9.**
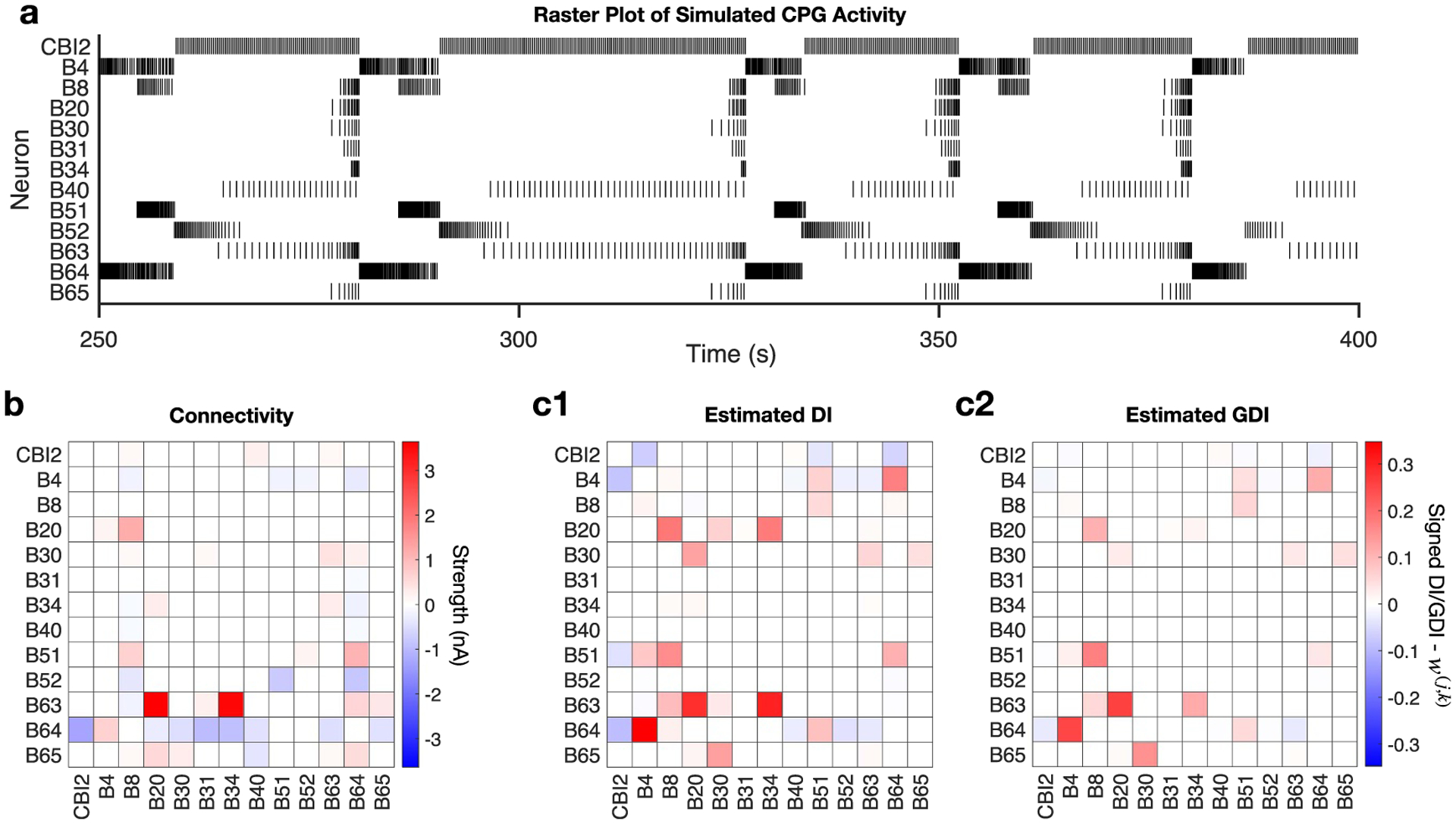
Connectivity inference on simulated CPG network. (a) Raster plot of spike times resulting from simulation of CPG implemented in SNNAP. (b) Connectivity matrix for CPG network where values indicate the nA shift in rheobase. Positive values indicate a reduction in rheobase (excitatory connection) whereas negative values indicate an increase in rheobase (inhibitory). (c1) Non-graphical DI inference of CPG network connectivity. Many true connections are detected, however a variety of false positives are included particularly in the approximately top-left quadrant. (c2) GDI inference of CPG network connectivity. Many of the values representing false positives in the DI analysis are correctly reduced while the stronger true connections generally seem to be appropriately preserved.

As further exploration of the use of GDI in inference of neuronal network connectivity via binned spike times, we move from an arbitrary network to a model of a CPG circuit in *Aplysia*, which mediates feeding behavior [40]. Figure 9(a) shows a raster plot of the spikes generated with SNNAP for individual identified neurons, highlighting the periodic and bursty nature of this circuit. B31, B51 and B64 were modeled with two compartments. Only the activity in the axonal compartments were examined by DI and GDI. In total, 400 s of data was simulated and used to perform the analyses. Network activity was elicited by simulated constant current injection of 1.3 nA into neuron CBI2 starting at 2 s to the end of the simulation. Figure 9(b) displays the synaptic connections present within the CPG model. Many of the CPG synaptic connections are complex, for example containing an early excitatory component followed by slow inhibitory component. Therefore, the physiological synaptic strength does not correspond linearly with the value of the synaptic conductance. Consequently, the strength of the synaptic connections was measured by the shift in the minimum current necessary to elicit a spike in the postsynaptic neuron (i.e. rheobase) during presynaptic spike activity. The rheobase was determined by delivering a stepwise series of current pulses (0.035–0.1 nA) with each successive step having a duration of 250 ms and a total duration of 8.5 s. The procedure was repeated during a 4 Hz, 8.5 s train of spikes in the presynaptic neuron elicited by a train of depolarizing current pulses (4 Hz, 30 ms, 12 nA) while the potential of all other neurons was held at −60 mV. This stimulation protocol was performed for each neuron pair with a 20 s interval between each measurement.

In order to estimate the connectivity of the network from the simulated spike times, we must take into account that the network incorporates both rapid and slow, traditional and modulatory synapses. Accordingly, although we fix *M* = 3 in order to keep dimensionality low, we estimate DI and GDI across a variety of bin widths ranging from 15 to 40 ms in steps of 5 ms, meaning that the history length accounted for ranges from 45 to 120 ms. The resulting DI and GDI values are then averaged across bin widths to produce the estimates shown in figure 9(c1) and (c2). Estimates for individual bin widths used 50 bootstrap iterations of splitting the data into training and testing sets for the underlying classifiers. As with the prior subsection, the DI values associated with a particular bin width were normalized and then set to zero if less than 0.01 [1], and the corresponding GDI estimates only conditioned on nodes having a DI value with the target node that was greater than or equal to 0.01. Sign inference is performed for each bin width, and then an average is taken to infer the sign overall. Specifically, for a given connection the signs are computed at the bin widths that produce non-zero DI/GDI estimates, and then the sign of the average of the individual sign values is used. Note again that our DI and GDI results use TLC and TLPC for sign inference, respectively.

The non-GDI estimates averaged across bin widths (figure 9(c1)) do identify many of the true connections, such as B63→B20, B63→B34, B51→B8, B64→B4, and B20→B8. However, a fair number of false positives were also identified. In particular, DI incorrectly identifies connections B30↔B20, B20→B34, CBI2↔B4, B51→CBI2, and B51→B4 (figure 9(c1)).

GDI averaged across bin widths (figure 9(c2)) generally preserves the stronger true connections to some degree while reducing many of the false connections incorrectly identified in the DI analysis. For example, the strong true connections B63→B20, B63→B34, B51→B8, B64→B4, and B20→B8 returned large GDI values (figure 9(c2)). Whereas DI incorrectly identified connections B30↔B20 and B20→B34 (figure 9(c1)), GDI successfully minimized these false connections (figure 9(c2)). Furthermore, the incorrectly inferred mutual inhibition between CBI2 and B4 by DI has been practically eliminated by GDI. Similar success was achieved by GDI for the false connections from B51 to CBI2 and B4.

We do note that the strong true connection from B51 to B64 is detected by DI but significantly reduced by GDI. Notably, the activity (figure 9(a)) of B64 and B4 overlap completely, whereas B51 and B64 are visually less related. For example, B51 does not fire during the final burst shown of B64 (figure 9(a)). The GDI value for B51→B64 is therefore fairly low while the value for B4→B64 is still reasonably high. This clear (but not synaptically-induced) pairing between B4 and B64 also likely contributes to the significant reduction incurred by GDI upon the strong true connection B64→CBI2. Because GDI captures unique influence between neurons, it does not significantly differentiate whether B4 or B64 is in fact synaptically inhibiting CBI2.

## 4. Discussion

DI measures of functional connectivity have been attractive because of their ability to make model-free causal inferences that account for nonlinear relationships. In neuroscience, DI and the related transfer entropy have become increasingly popular [10–12]. However, a long-standing issue that has been faced by DI has been the conditioning of inferences on more than one other node’s activity without model assumptions. In network neuroscience, this issue is of fundamental importance because being able to condition on all relevant nodes is critical to making the distinction between direct and indirect connections between brain regions or individual neurons [41]. Previous methods have been unable to achieve such wide-scale conditioning because of their poor scaling performance with regards to dimensionality, which increases with the inclusion of more and more nodes in conditioning.

Our GDI results show that recent advances [13, 14] allow one to solve high-dimensional problems with good scaling performance and thereby significantly address indirect connectivity issues common to connectivity analysis. Although we show strong performance for a linear and Gaussian network, the performance of GDI on a nonlinear network involving uniform random variables is notable and of significance for neural recordings which are unlikely to be simply linear and Gaussian. GDI also exhibits excellent conditioning performance on networks with conductance-based realistic neurons, particularly in its ability to detect connections while ruling out numerous indirect connections in a model of the CPG in *Aplysia*. These simulations not only highlight the ability of GDI to characterize many-node networks but also reveal another unique feature of our framework: GDI is universal in that it can be applied both to continuously-valued and discretely-valued problems. Therefore, GDI can be used on data types ranging from binned spike times to voltage recordings such as ECoG or EEG. One limitation of our study is that GDI was not applied to such real data, and therefore an immediate objective of future work should be to use GDI on actual recordings.

The GDI approach also provides a sign inference methodology that conditions on the activity of other nodes unlike some prior connectivity inference methods [1, 30]. These prior approaches only look at pairwise interactions for sign inference, which falls short of the multivariate inference that one can perform in a network of nodes. We show through examples that there are conditions where using solely pairwise methods for sign inference will mistakenly identify the sign of connections, such as labeling inhibitory connections as excitatory. By using our partial correlation approach, we are able overcome this limitation by accounting for the contributions of other nodes, which in our example leads to correctly identifying the aforementioned connection as inhibitory.

We close our discussion by commenting on two issues, namely edge selection and connectivity inference from circuit activity that exhibits patterns. The first issue of edge selection (i.e. choosing or determining which edges to retain or which edges are significant) is an issue faced in any type of graph analysis resulting from simulated or real data. Correlations or DI values between nodes are often non-zero even when there is no relationship between them. Such values may be low or infinitesimal, allowing the setting of an arbitrary threshold. Alternatively, one may have *a priori* knowledge of the network topology and can therefore select an appropriate number of connections to retain. However, we ultimately desire a data-driven approach to the edge selection problem, and we emphasize that this is an open area of research. Although progress has been made [42] on data-driven solutions for particular cases, we hope future work will shed more light on more general solutions.

The second issue of connectivity inference in pattern-exhibiting circuits has been commented on recently [43]. One key challenge is that when patterns occur in the activity generated by a neural circuit, activity is likely to be correlated or related (as measured by DI) even between neurons without direct connections simply because of the patterns. This is clear in the activity resulting from the CPG model (figure 9(a)) which results in many incorrectly identified connections by non-GDI (figure 9(c1)). Although GDI is able to reduce some of these indirect connections, it also reduces some true direct connections because of the significant overlap in neuronal activity (figure 9(c2)). Importantly, this highlights another key challenge faced in pattern-generating circuits, which occurs when one of two neurons (A and B) directly influences a third neuron (C) but neurons A and B happen to be overlapping in their behavior as a product of the generated pattern. Partial inference techniques such as GDI capture the unique influence between neurons, and if neurons A and B do not differ significantly in their behavior, then each of their influences on neuron C conditioned on each other may approach zero. Therefore, the direct connection from A to C could be missed by GDI. A pairwise method such as DI will identify A and B as influencing C because unique influence is not considered in pairwise analyses.

Overall, GDI reveals which neurons appear to be uniquely influencing other neurons by considering the rest of the network, while pairwise DI reveals which neurons appear to be influencing other neurons regardless of the activity of the rest of the network since activity outside of pairs of neurons is not considered by non-GDI. Therefore, in pattern-based circuits pairwise DI is susceptible to false positives while GDI is susceptible to false negatives. It should be emphasized that this is not a failure of the methods, but rather is due to the larger issue of being limited by the information present in activity generated by neural circuits and how that information relates to underlying anatomical connectivity. Circuits that do not generate patterns are expected to exhibit more variable activity among neurons, and GDI will use this variance to correctly identify direct connections and reduce indirect connections. To summarize, pattern-generating circuits are challenging for connectivity inference because neurons may behave very similarly, resulting in false pairwise inferences as well as false partial inferences.

## Data Availability

Programs and data files of this study are openly available at URL/DOI: github.com/jy46/GDI (DOI: https://doi.org/10.5281/zenodo.4207536).
